# Newly diagnosed high-risk localized prostate cancer after multiple [177Lu]Lu-PSMA radioligand therapy in the context of metastatic parotid carcinoma: a case report

**DOI:** 10.1186/s13256-026-06225-2

**Published:** 2026-07-06

**Authors:** Christopher Orf, Analena Elisa Handke, Joachim Noldus, Florian Roghmann, Martina Dellino, Karl H. Tully

**Affiliations:** Department of Urology and Neurourology, Ruhr-University Bochum, Marien Hospital Herne, Hölkeskampring 40, 44625 Herne, Germany

**Keywords:** Neoadjuvant PSMA-radioligand therapy, High-risk prostate cancer, Parotid carcinoma

## Abstract

**Background:**

Prostate-specific membrane antigen-direct radioligand therapy (PSMA RLT) is primarily used to treat castration-resistant metastatic prostate cancer (PCa) after prior treatment options have been exhausted. Occasionally, other tumor entities, such as parotid carcinoma in this case, also express prostate-specific membrane antigen (PSMA) on their cell surface.

**Case description:**

We present the case of a white 67-year-old patient newly diagnosed with locally advanced PCa despite multiple cycles of Lutetium-177-labeled prostate-specific membrane antigen radioligand therapy ([177Lu]Lu-PSMA-RLT), which he had received for metastatic parotid carcinoma. Interestingly, no regressive histopathological changes were found in the prostate biopsy specimen, despite given PSMA-expression in the prostate.

**Conclusion:**

The potential selection of high-grade disease, which appears to have occurred in this patient, underscores the need for further large-scale histopathological examination and thorough follow-up of the current investigational approaches using [177Lu]Lu-PSMA-RLT in high-risk prostate cancer (PCa).

## Highlight Box

**Key findings**

- A high-risk prostate cancer was diagnosed after multiple cycles of [177Lu]Lu-PSMA-RLT, conducted due to a known parotid carcinoma. Moreover, no regressive histopathological changes were found in the prostate biopsy specimen, indicating a potential selection of high-grade disease.

**What is known and what is new?**

- [177Lu]Lu-PSMA-RLT RLT is primarily used as a further line treatment regimen of castration-resistant metastatic prostate cancer
- Occasionally, [177Lu]Lu-PSMA-RLT is used for the treatment of parotid carcinoma as well. Despite using the exact dosages and application modality, a high-risk prostate cancer ensued.

**What is the implication, and what should change now?**

- Neoadjuvant therapy using [177Lu]Lu-PSMA-RLT in high-risk prostate cancer may become a valuable tool in the treatment cascade of localized PCa, but needs further investigation, especially concerning reliable predictive biomarkers conferring resistance to [177Lu]Lu-PSMA-RLT

## Introduction

Currently, Lutetium-177-labeled prostate-specific membrane antigen radioligand therapy ([177Lu]Lu-PSMA-RLT is primarily used to treat castration-resistant metastatic prostate cancer (PCa) after other treatment options have been exhausted. In some cases, other tumor entities, e., parotid carcinoma, also express PSMA on their cell surface. The same therapeutic prostate-specific membrane antigen (PSMA) ligand radiolabeled with Lutetium-177 (177Lu) is used for both types of tumors. Through this retrospective analysis, we aim to highlight that the application of multiple cycles of [177Lu]Lu-PSMA RLT in parotid carcinoma in this particular case did not result in any significant histopathological regression in the prostate biopsy specimens. This aspect might be interesting as [177Lu]Lu-PSMA-RLT is under investigation as neoadjuvant therapy approach in localized PCa [9].

## Case presentation

**Fig. 1:**
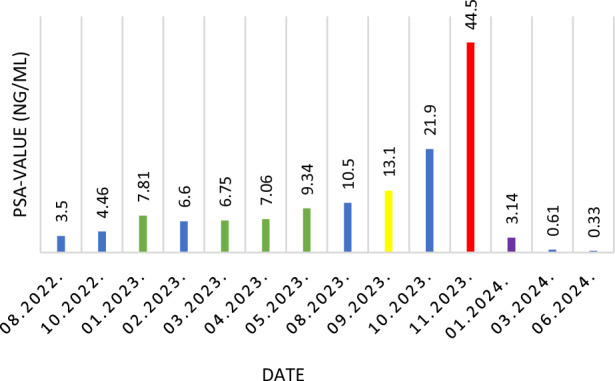
PSA progression: the PSA value of 13.1 ng/ml (in red) represents the level at the time of prostate MRI, while the PSA value of 44.5 ng/ml (in orange) was measured at the time of the prostate biopsy. Values in green are those under PSMA-RLT. Further values starting 01/2024 show a decrease under further PCa therapy

A 67-year-old white patient presented to our outpatient clinic due to a steady increase in his prostate-specific antigen levels (PSA) for further diagnostic workup. The current blood work showed a PSA level of 21.9 ng/ml. Previously, PSA levels had been increasing over the last 7 months (Fig. 1). The digital rectal examination and transrectal ultrasound revealed no pathologic findings. The patient presented to our urology clinic in good general and normal nutritional condition. The patient was wearing a corset due to a pathologic fracture of the fifth right lumbar vertebral arch, caused by bone metastases of right-sided parotid carcinoma. There were no other known comorbidities. Based on the current urological findings, a prostate biopsy was scheduled.

**Graph 1:**
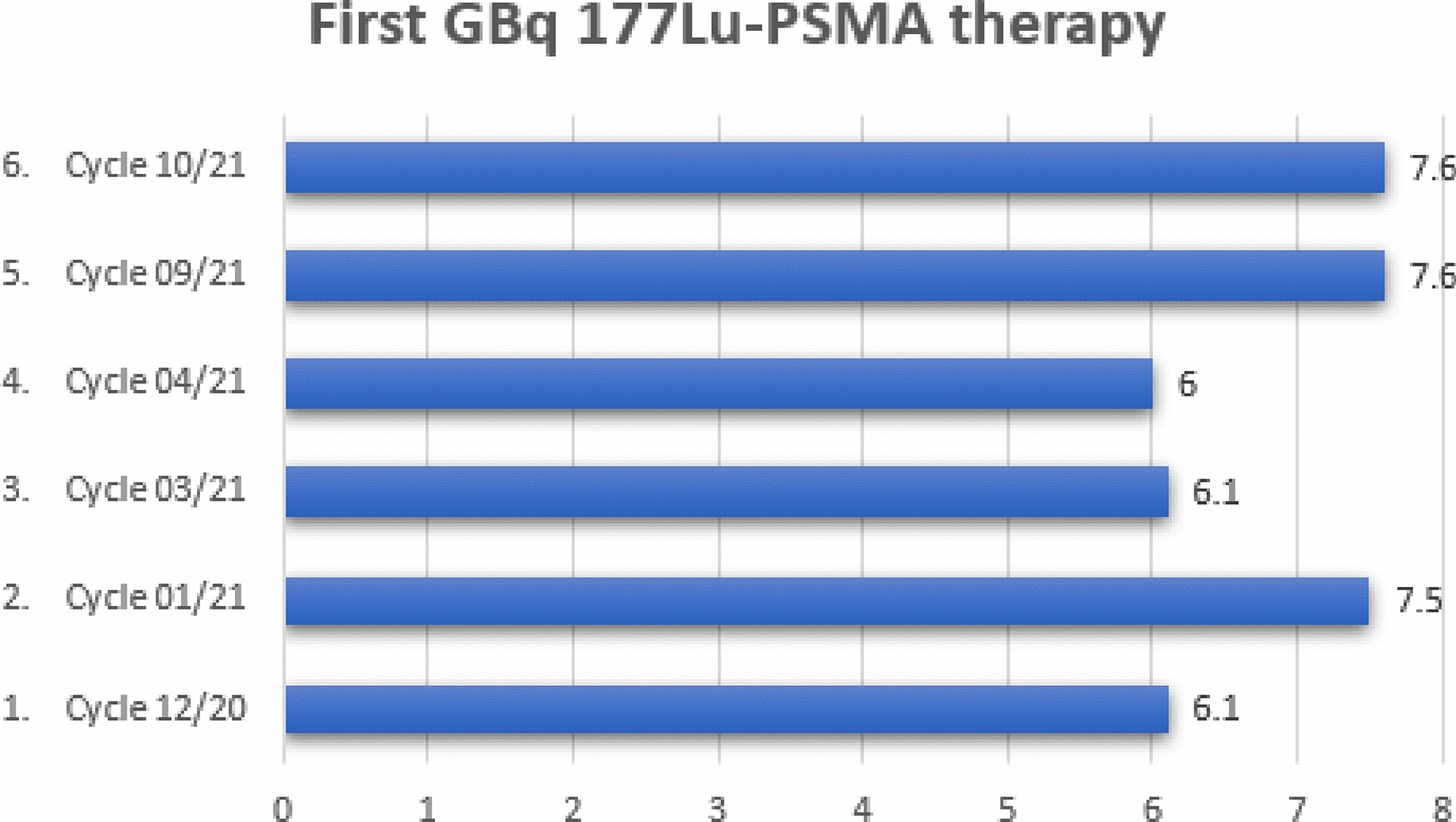
Dosage in GBq of the first radioligand therapy

**Graph 2:**
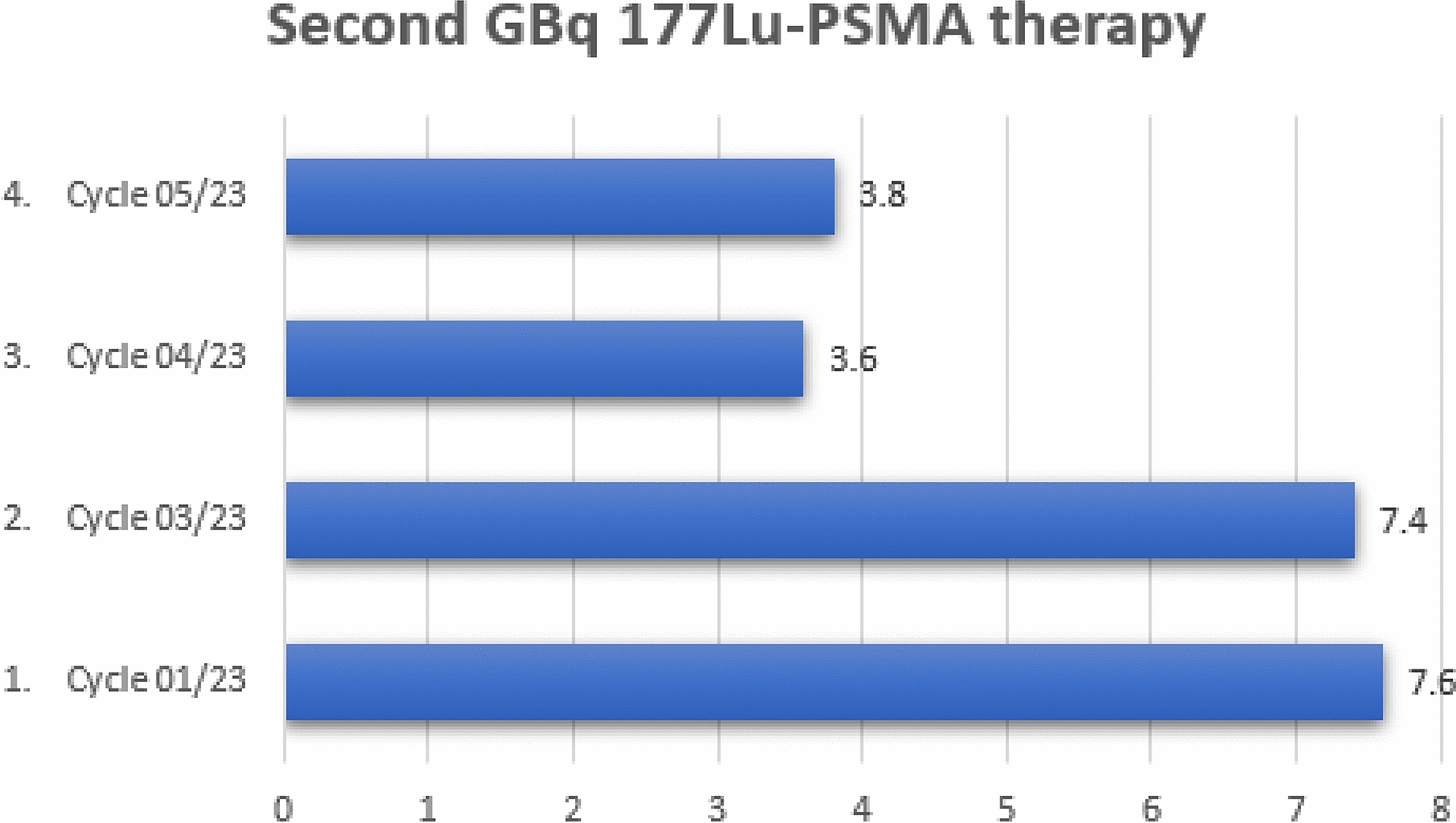
Dosage in GBq of the second radioligand therapy

Regarding the history of parotid carcinoma, the patient first underwent removal of the right parotid gland in 2004, followed by neck dissection in 2010 due to local tumor recurrence. Subsequently, ten rounds of [177Lu]Lu-PSMA RLT with a total of 64,3 GBq [177Lu]Lu-PSMA-617 (Graph 1 and 2) and an external irradiation (30 Gy) of lumbar vertebral metastases had been performed from 2020 to mid-2023. The status of the osseous metastases had been previously documented several times using positron emission tomography with computed tomography (PET/CT) after each cycle of [177Lu]Lu-PSMA RLT. A constant number and size of disseminated, increasingly sclerotic bone metastases with moderate to intense PSMA expression was considered a therapeutic response.

**Fig. 2:**
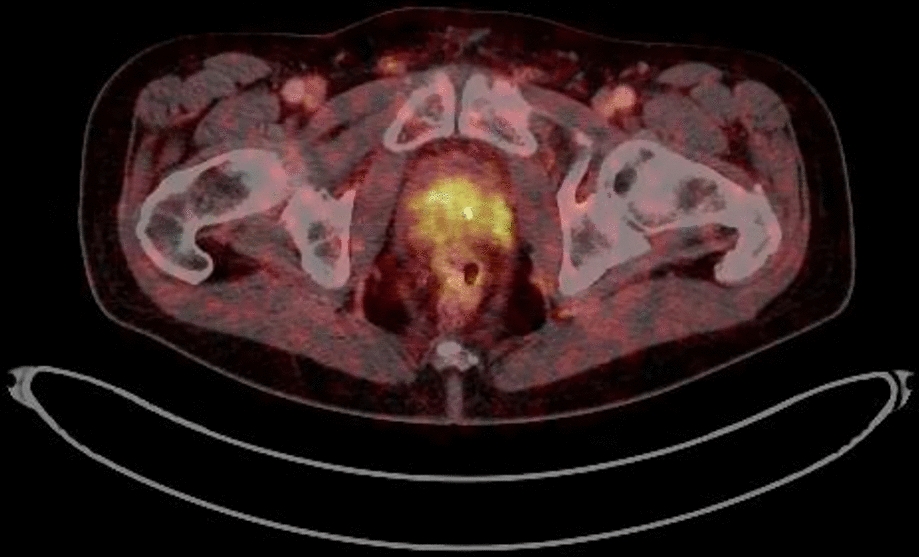
PSMA-tracer uptake of the prostate gland

**Fig. 3:**
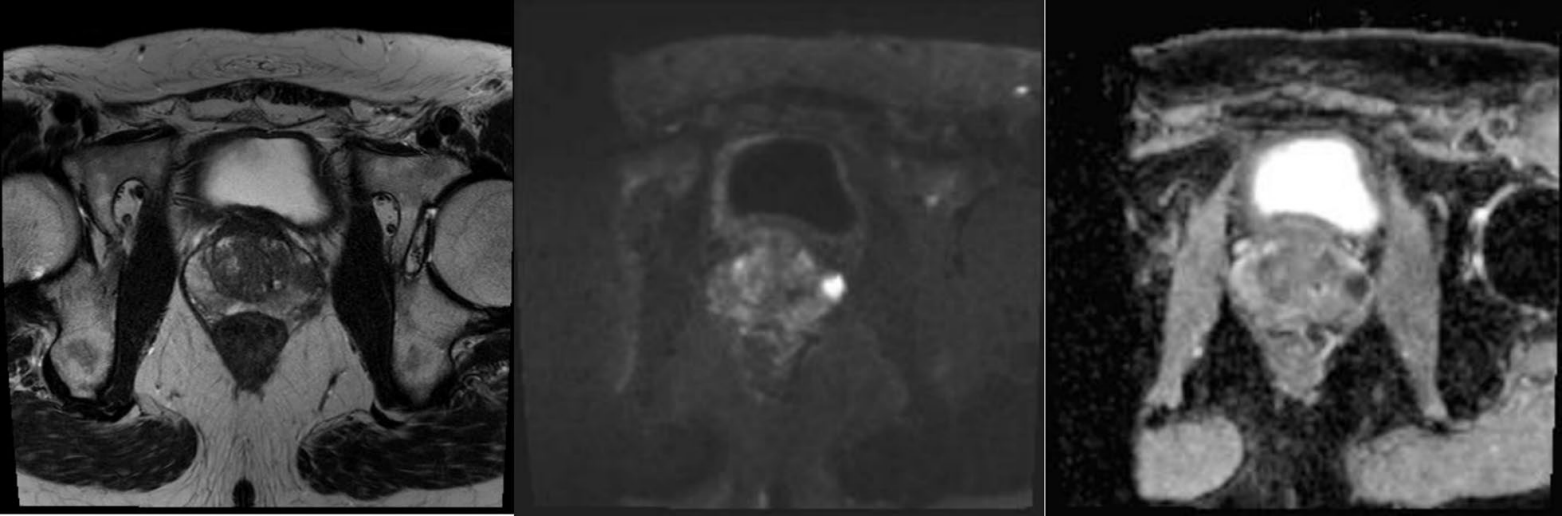
PIRADS-4 lesions in the peripheral zone postero-lateral and anterior (PZpl/PZa) and apical peripheral zone postero-medial (PZpm) on the left side

During one of the regularly scheduled imaging examinations for parotid carcinoma in 2023, a new, high uptake of the PSMA-tracer was observed in the prostate (Fig. 2). A follow-up examination using multiparametric magnetic resonance imaging (mpMRI) of the prostate was performed later the same year revealing two PI-RADS-4 lesions on the left side of the prostate (Fig. 3), as per the Prostate Imaging Reporting and Data System (PI-RADS) Version 2.1 criteria.

A few weeks before the scheduled prostate biopsy, the patient was admitted to the hospital due to intermittent right leg paresis and alternating pain and tingling paresthesia. Guided by his medical history, the patient underwent comprehensive neurological and radiological diagnostics (computed tomography (CT) of the cranium and the entire spine), revealing new bone metastases affecting the whole spine, and both hips and scapulae. Due to this newly diagnosed increase in the patient´s metastatic volume and simultaneous elevated PSA value, it was unclear whether this was either due to a progressive disease of the known metastases of the parotid carcinoma or to a de novo metastatic PCa.

**Fig. 4:**
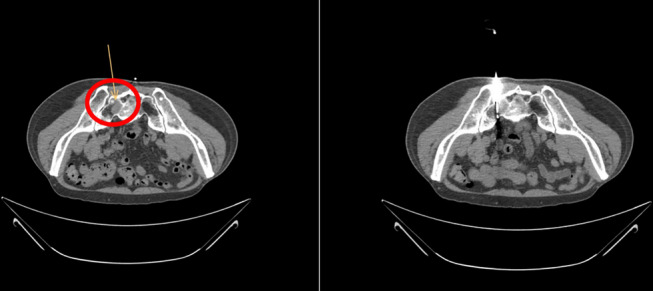
Biopsy of the metastasis on the left sacral bone

**Fig. 5:**
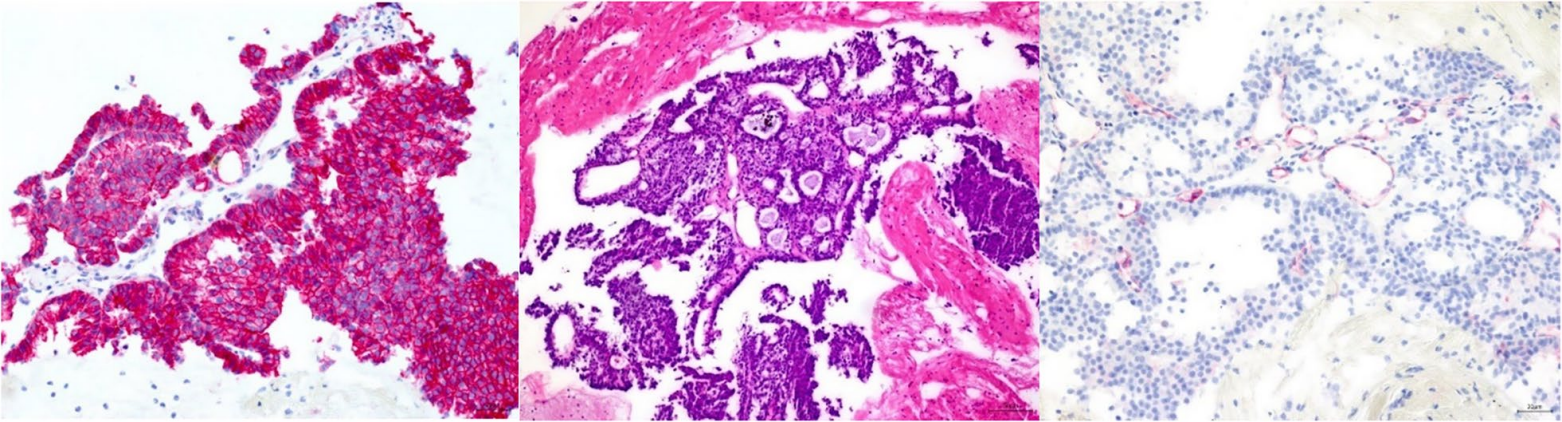
Bone metastasis with small tumor cell clusters of an acinar cell carcinoma showing typical histology and viable tumor cells. No regressive changes were observed, including no decrease in tumor cellularity, no stromal fibrosis, and no extracellular mucin deposits. The assessment of regression is limited due to the presence of only isolated tumor cell clusters

Following his discharge from the neurological department, he underwent a transperineal mpMRI fusion biopsy. A 12-core systematic and two three-core targeted biopsies of both PIRADS 4 lesions (Fig. 3) were conducted. Pathologic review revealed a high-grade PCa (see below “histopathological findings”). The previously conducted CT imaging studies were then further reviewed to plan a biopsy of the osseous lesions to differentiate between metastatic lesions of the newly diagnosed prostate and parotid carcinoma, respectively. A biopsy of a morphologically new lesion in the sacral bone was performed (Fig. 4). Histologically, a metastasis of the previously known parotid carcinoma was found (Fig. 5).

### Histopathological findings

**Fig. 6:**
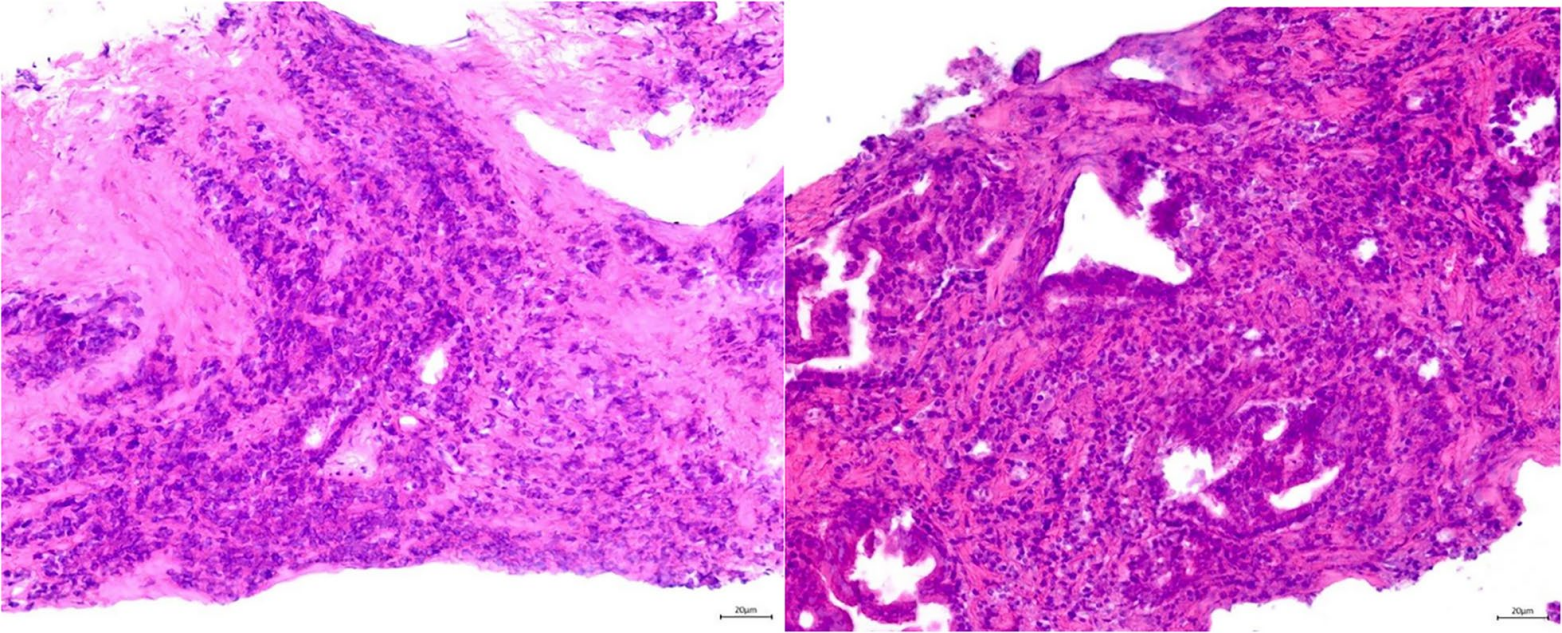
Biopsy cores of the transperineal MRI-guided prostate biopsy with Gleason Grade 4 and Gleason Grade 5 pattern showing no significant regressive changes

In the biopsy cores of the transperineal MRI-guided prostate biopsy, six out of 18 prostate biopsy cores showed a PCa (infiltration between < 5 and 40%), Grading G3, Gleason 9 = 4 + 5, World Health Organization (WHO) grade 5. Notably, no regressive changes were observed after [177Lu]Lu-PSMA-RLT for parotid carcinoma (Fig. 6). We discussed additional immunohistochemistry with our pathologists. We have decided by consensus to focus on histopathologic alterations as the majority of studies use imaging methods or PSA rather than molecular targets.

### Therapy

After histopathological confirmation of PCa and histological evidence of a tumor of the previously diagnosed acinar cell carcinoma, the case was presented in our interdisciplinary tumor conference. Based on the limited prognosis and the missing evidence of metastatic PCa, a multidisciplinary consensus was reached to start androgen deprivation therapy (ADT).

### Follow-up

Subsequent PSA values from 2024 remained high under ADT (3.14 ng/ml). Consequently, the decision was made to initiate additive therapy with Enzalutamide. The following PSA values showed declining levels at 0.33 ng/mL. The patient died of progressive metastatic parotid carcinoma in 2024.

## Discussion

PSMA is a membrane-bound glycoprotein predominantly expressed in prostate cells. PCa cells increasingly express PSMA on their surface by 100–1000-fold [1, 2]. Therefore, they are easily accessible in nuclear medicine procedures, such as PSMA PET/CT, and/or novel therapeutic approaches, such as [177Lu]Lu-PSMA-RLT. Today, [177]Lu-PSMA-RLT is primarily used to treat castration-resistant metastatic prostate cancer after prior treatment options have been exhausted. Occasionally, other tumor entities, such as parotid carcinoma in this case, also express PSMA on their surface. The same therapeutic PSMA ligand Lutetium 177 is used for both tumor entities. [177]Lu is a medium-energy *β*-emitter and low-energy gamma emitter with a physical half-life of 6.73 days and a mean path length of 2 mm. Due to its properties, it allows sufficient irradiation to both large and small tumors, thus optimizing the biological effect and causing less toxicity [3, 4]. [177Lu]Lu-PSMA will be internalized and held within the tumor cells and is known to cause few adverse events [5, 6]. Ahmadzadehfar et al. noticed no side effects immediately after injection [7]. Anemia, leukopenia, and thrombocytopenia occurred in one case seven weeks after radioisotope administration, with no relevant nephrotoxicity or hepatotoxicity noted [7]. Non-hematological side effects, such as fatigue, nausea, and low-grade Xerostomia, may also appear [8].

Moreover, [177Lu]Lu-PSMA RLT is under investigation as a possible neoadjuvant therapy approach, as displayed in the”LuTectomy” trial published by Eapen et al. [9]. In this pivotal trial, the investigators examined the radiation dose absorbed by the tumor (primary endpoint), adverse events, surgical safety at the time of prostatectomy, as well as imaging and biochemical responses in 20 patients receiving either one or two cycles of [177Lu]Lu-PSMA-RLT in a neoadjuvant setting followed by radical prostatectomy. ADT was not prescribed in any setting of the trial. They observed a PSA decline of more than 50% in nine (45%) patients. Moreover, only a few toxicities were observed, including grade 1 fatigue in eight (40%), nausea in seven (35%), dry mouth in six (30%), and thrombocytopenia in four (20%) patients [7], respectively. There was no impact on surgical safety, as no grade 3 or 4 toxicities or Clavien-Dindo 3–5 complications occurred [9]. Further Phase I and II trials support the safety and general feasibility, as well as efficacy, of neoadjuvant [177Lu]Lu-PSMA-RLT in terms of PSA reduction and tumor downgrading [10]. However, long-term follow-up data are lacking to this day [11]. In the presented case, the patient was diagnosed with high-risk PCa following multiple cycles of [177Lu]Lu-PSMA RLT. This development suggests a potential selection process for PCa cells using [177Lu]Lu-PSMA RLT. This hypothesis is supported by the continuous progression of PSA over time under ongoing [177Lu]Lu-PSMA RLT. The LuTectomy trial observed high levels of targeted radiation doses in the prostate with pathological tumor regressions in 80% of patients [9]. Interestingly, no regressive alterations were observed in our patient. The final histopathological examination revealed no alterations in tumor cell density, stromal fibrosis, or extracellular mucin deposition, indicating no histologic regression. Regression patterns are expected to resemble those seen after RT or neoadjuvant systemic therapy. However, clinical data remain limited and are often based on preclinical data, which may be due to a lack of routine histopathologic sampling, as most clinical assessments rely on imaging and PSA response rather than tissue analysis [12, 13]. Considering a selective process where a certain subgroup of tumor cells is more resilient to [177Lu]Lu-PSMA, this should lead to regressive histopathological features to some extent, either by destruction of the less resilient tumor cells or by radioactive radiation itself. Alternatively, it must be assumed that the presented patient had high-risk PCa from the very beginning, and despite multiple cycles of [177Lu]Lu-PSMA RLT and confirmed PSMA expression, the tumor progression went on, indicating a possible resistance to [177Lu]Lu-PSMA-RLT, which can be observed in about one-fourth of patients [14]. Slootbeek et al. were able to show that TP-53 loss-of-function alterations in metastatic castration-resistant prostate cancer patients under [177Lu]Lu-PSMA-RLT are associated with a shorter progression-free survival [15], indicating a possible explanation for the tumor resistance in our case but questioning the concept of neoadjuvant [177Lu]Lu-PSMA RLT for all patients and warranting further research to improve patient selection for this potential new treatment strategy.

## Conclusions

The presence of PCa prior to the patient’s primary malignancy (i.e., his parotid tumor) cannot be fully ruled out in this case. Given the clinical course of the patient and his rising PSA and newly found prostatic tracer accumulation might indicate that there were unfavorable changes to the patient’s prostate cancer under [177Lu]Lu-PSMA-RLT. The potential selection of high-grade disease, which seems to have occurred in this patient, underlines that the current investigational approaches of neoadjuvant therapy using [177Lu]Lu-PSMA-RLT in high-risk PCa require further large-scale histopathological examination and thorough follow-up.

## Data Availability

Available for review by the Editor-in-Chief of this journal.
